# Delphi Survey of Items for the Test of Stuttering Screening in Children (TSSC)

**DOI:** 10.2174/17450179-v19-230615-2022-27

**Published:** 2023-07-26

**Authors:** Aiswarya Liz Varghese, Radish Kumar Balasubramanium, Gagan Bajaj, Sudhin Karuppali, Unnikrishnan Bhaskaran

**Affiliations:** 1 Department of Audiology and Speech Language Pathology, Kasturba Medical College, Mangalore, Manipal Academy of Higher Education, Manipal, Karnataka, India; 2 Department of Community Medicine, Kasturba Medical College, Mangalore, Manipal Academy of Higher Education, Manipal, Karnataka, India

**Keywords:** Stuttering, Screening, Delphi survey, School going children, Test, SLPs

## Abstract

**Purpose:**

Stuttering is a fluency disorder that mostly begins in childhood and affects many people in our societies. No standardized screening tools are available to check for stuttering in the Indian school-going population. Thus, the study aimed at developing a screening tool to identify children who stutter among the school-going population using a Delphi-based approach.

**Methods:**

This study was carried out in four phases. During the first phase, five Speech Language Pathologists (SLPs) were asked about the need for screening and the nature & attributes of a stuttering screening test for school-going children. The second phase involved constructing appropriate stimuli for the screening tool based on expert opinion, relevant literature and students’ academic textbooks. The third phase involved content validation of the speech elicitation stimuli by four teachers, five SLPs and an English Lecturer teaching in a university. The fourth phase encompassed the development of differential diagnosis criteria for stuttering identification in children using a rank analysis of the expert opinions.

**Results:**

A stuttering screening stimuli comprising age, language and culture-specific reading, picture description and narration tasks for 1^st^ to 10^th^-standard students was developed. The contents of the tool obtained satisfactory consensus of agreement among the panel of experts. Further, the tool outlined five critical diagnostic criteria which could differentially diagnose school-going children with stuttering from typically speaking counterparts using the developed material.

**Conclusion:**

The developed screening tool could help practicing clinicians quickly identify stuttering in school-going populations. This would enable early identification and build up the statistical data to estimate the prevalence of stuttering among the school-going population. Further studies examining the psychometric properties of the developed test are in progress.

## 1 INTRODUCTION

Stuttering is a fluency disorder that mostly begins in childhood affecting a large number of people in society. Earlier studies in the twentieth century have reported a prevalence rate of stuttering ranging from 0.35% to 2.61% in English-speaking American and British children [1-15]. A person who stutters will have involuntary disruptions in their speech fluency, consisting largely of syllable repetitions, prolongations, blocking of sounds, substitutions and avoidance of words [16, 17]. Normal-like brief disfluencies, such as phrase repetitions and interjections (use of words like ‘I mean” or “umm”), are considered acceptable and not necessarily a problem [18, 19]. However, these normal disfluencies pose challenges in diagnosing children with stuttering, especially during their early critical years of language learning. Nevertheless, it is important to evaluate if there are chances that these normal disfluencies in communication continue into their later life. Identification of such children at risk for stuttering supports early treatment services at a young age where chances for improvement are at their best [20]. This rationale supports the stuttering screening among school going population.

From the studies reviewed, it is clear that most large-scale studies screening school-going children have used questionnaires returned by teachers/informants to identify stuttering [21, 22]. Only a few studies have used a direct face-to-face examination of children with stuttering [23], where the point prevalence of stuttering was about 1%. Some studies have used a combination of questionnaires returned by teachers followed by face-to-face examinations of children with stuttering [24]. Moreover, the existing standardized tools for diagnosing stuttering, such as Stuttering Severity Instrument (SSI) [25], are time-consuming, and the stimuli are not standardized for the Indian population. Also, no standardized screening tools are available to check for stuttering in the school-going population. Keeping these aspects in mind, the authors felt a need for a tool to screen Indian school-going children with stuttering. Hence the present study was planned with an aim to develop a screening tool to identify children who stutter among the school-going population. The three main objectives of the study included the construction of a tool to screen stuttering among school-going children, content validation of the constructed tool, and formulation of the diagnostic criteria to differentially diagnose stuttering from normal speech disfluencies using the developed tool.

## 2 METHODS

The present research was approved by the Institutional Ethics Committee. (Approval No: IEC KMC MLR 03-18/44). The stimuli for each task and interpretation of test findings were developed and validated across four phases using a Delphi survey-based approach [26]. The Delphi method anonymously gathers expert opinions through a series of questions and analysis techniques, with controlled feedback in an iterative process. The key strength of the Delphi method is the objective survey of contents that require judgement while developing a clinical assessment instrument. This design was adopted because the Delphi method is considered one of the most commonly used research procedures to establish the content validity of an assessment instrument by an expert panel. The first phase focused on the ‘Needs Assessment’ followed by constructing the various attributes of the test, like the stimuli and tasks, during the second phase. The third phase involved the content validation of the constructed tool, followed by the fourth phase, wherein consensus for the diagnostic criteria for the developed tool was established. The methodology here further describes each of the four phases in detail.

### 2.1 Phase 1

The first round of the Delphi survey began wherein a panel of five speech-language pathologists (SLPs) with more than ten years of clinical and research experience in the domain of fluency disorders were contacted. All the experts were informed about the study's purpose, expected time commitment, participation criteria, and the need to stay involved throughout the research process. They were also told that their participation in the Delphi study was voluntary. After that, written consent was obtained from all the experts. Each participant who agreed to participate was given a unique code to protect their confidentiality and anonymity. Each expert was asked for their preferred mode of receiving the Delphi round questionnaires (either printed or mailed versions), and contact was established with them accordingly. The demographic characteristics of these SLPs are provided in Table **1**.

The SLPs, during this phase, were asked to provide their opinion about three questions, *i.e.*, “Whether they feel that there is a need for a screening test for quick identification of stuttering in their clinical practice with school-going children,” “What advantages would be there if such a test is available” and “What should be the nature of speech elicitation tasks in one such tool for the school going children.” The professionals were given a span of two weeks to answer the questionnaire, with a reminder sent during that time. The researchers analyzed the answers qualitatively received from the professionals. A summary of the results was then shared with the participants as controlled feedback to inform them of other participants' points of view and offer them a chance to clarify or modify their opinions during the Delphi procedure. The collective feedback from all the experts was gathered to support the development of screening tests for children and determine the speech elicitation stimuli based on the review of the relevant literature in this domain [25]. The suggestions made by the expert panel during this phase, reading, spontaneous speech tasks and picture description, emerged as the key speech elicitation tasks relevant to the school-going children; therefore, phase 2 focused on the construction of these tasks.

### 2.2 Phase 2

During this phase, the researcher reviewed the textbooks from the 1^st^ – 10^th^ standards to obtain the repertoire of words used in each standard. The most frequently occurring words from the language textbooks of each standard were included in the word list. A common theme of stringing the words was picked up for each standard, and reading passages were made for 4^th^ -10^th^ standards using the selected words from textbooks of their respective standards. Similarly, picture cards were made available for 1^st^-3^rd^ standards from the respective wordlists since these students were still acquiring reading proficiency and describing a picture card was easier than reading. A common theme stringing the obtained word list was finalized to be represented in a series of events in a single picture card, and it was digitally depicted with the professional help of a technical expert. The third stimulus used for screening was a spontaneous speech task where the topic for each class was selected from the repertoire of words obtained from textbooks of each class. The topic was selected so the children could spontaneously talk about a theme using age-appropriate vocabulary. Likewise, three picture cards were determined for the first, second and third standard students, three reading passages each for fourth to tenth standard and three topics each from first to tenth standard. A sample of these constructed stimuli has been described in the results section.

### 2.3 Phase 3

The third phase involved content validation of the constructed passages, picture cards and topics of spontaneous speech tasks. Five Speech Language Pathologists, four teachers and one English professor affiliated with a university teaching college were approached to validate the stimuli for each task. The demographic details of these participants are shown in Table **2**. These words were also approved by the teachers of the respective standards and observed to be readable by the students of those standards.

Teachers were given the passages so that they could check whether it was readable by the students. The passages were content validated by an English Professor teaching in a University affiliated college, and modifications were incorporated accordingly. Five Speech-Language Pathologists also were asked to validate the content based on the readability of the stimuli, vocabulary and grammatical appropriateness, the flow of information, and cultural adequacy for each standard. Participants rated the developed stimuli using the questionnaire developed for this purpose. The content-validated questionnaire which was circulated is attached in Appendix A. A quantitative and qualitative analysis of the outcomes from this round of the Delphi process was conducted. A threshold of 80% was used as the agreement standard, and the consensus level achieved was calculated. Any items that received agreement from more than 80% of participants were kept, while those with less than 20% agreement were removed. For items with agreement between 80% and 20%, the researchers reviewed ratings and comments to make revisions. The revisions continued until all the items reached the desirable consensus on the agreement.

### 2.4 Phase 4

Participants for phase IV included ten SLPs working in the field of fluency disorders with similar inclusion criteria as the one followed during the first phase. The expert panel in the fourth phase differed from Phases 1 and 3. The correspondence with these experts was done similarly during the first phase, and informed consent was obtained before their participation. The demographic details of these participants are shown in Table **3**.

These 10 participants were contacted independently per their convenient mode of correspondence and asked to list the ten most critical differential diagnostic features in a ranked manner, which they use to differentiate stuttering from normally occurring disfluencies among school-going children. All the responses of the SLPs were tabulated and analyzed to obtain the features which the panelists agreed upon in descending rank order.

Once the content of the developed tool reached a satisfactory mark and the diagnostic criteria received the consensus, the Delphi procedure was terminated, and the results were shared with the expert panel with an acknowledgement note.

## 3 RESULTS

The present study aimed to develop a screening tool to identify children who stutter among the school-going population. The three main objectives of the study included the construction of a tool to screen stuttering among school-going children, content validation of the constructed tool, and formulation of the diagnostic criteria to differentially diagnose stuttering from normal speech disfluencies using the developed tool through a Delphi process. The results of each of the four phases have been discussed below.

### 3.1 Phase 1

During the first round of Delphi, the expert panel comprising five SLPs was asked initially about the need for developing a screening test for stuttering. The results revealed that 100% of these panel members agreed to develop a test to screen stuttering among school-going children. The factors that were determined to help create a screening test included impartiality in screening, using consistent and appropriate stimuli for the Indian population's culture, and the availability of adequate time and personnel to screen a large number of children in their school surroundings. When asked about the nature of speech elicitation tasks in one such tool for the school-going children, the panel recommended the usage of picture cards for those who had not yet achieved adequate reading and writing skills, as in the first and second standard students; reading passages for the students between third to tenth standard; and a spontaneous speech task with a topic relevant to the age and stage of the child. The panel further emphasized that these picture cards, reading passages and topics for spontaneous speech should be determined to be appropriate to the Indian culture and incorporate the vocabulary appropriate to the given child’s language age.

### 3.2 Phase 2

The content and item selection for the screening test of stuttering was based on theoretical constructs related to the development of fluency skills and the opinions of the experts during the first phase. The three sections of the screening test considered were Picture description, reading and spontaneous speech tasks. In line with these suggestions, an initial list of words selected was from the school-going children's academic textbooks. These word lists were analyzed, and the frequently occurring content words were used to construct a reading passage. Reading passages were constructed using these words with the appropriate prepositions and conjunctions to connect the words. The sentences for the reading passage were constructed in such a way as to give a connected flow of information on a particular topic. The number of words selected from the textbooks for each standard, with examples of some of the chosen words and constructed sentences, have been provided in Table **4**.

Per the expert panel's suggestions, reading activity was not considered for children in 1^st^ -3^rd^ standards. Picture description task was thus used instead of reading passages for these children. Picture cards were digitally created, depicting a theme consisting of words chosen from their textbooks. Three picture cards were digitally created for each standard based on the increased opportunities to talk from the available pictures. Three to four target words from their textbooks were included in each picture task, and those words could be elicited during this task. A sample of some of these pictures is shown in Fig. (**1**).

Similarly, a set of 3 topics were selected from the word list generated from their textbooks for the purpose of spontaneous speech tasks. The topics were selected so that there are opportunities to discuss the same. The explanations of the topics are already provided in the textbooks, and it was selected under the pretext that children would have learned about them in the classroom. Of the 3 topics selected, each student was free to choose and talk about it based on their knowledge. A sample of these topics for each of the standards has been given in Table **5**.

### 3.3 Phase 3

The content validity of the proposed screening test was conducted by asking the expert panel about the relevance of each subsection of the screening protocol and finding out the coherence of each section with respect to the age and class of the student being tested. The questions consisted of the relevance of separate sections and the appropriateness of the stimuli used under each section. A set of close-ended questions were included in the content validity questionnaire. All the experts either agreed or strongly agreed on the questions pertaining to the significance and suitability of the subsections. The expert panel proposed recommendations on the addition or removal of items. Feedback from the experts’ panel on the screening tool was used to modify the overall contents of the test. The university English professor corrected the grammatical errors in certain reading passages. The school teachers also commented on including age-appropriate words for the reading task according to each standard. Only the stimuli either agreed or strongly agreed by 80% of the panel members were included in the final test. The modified version of the proposed test was mailed back to panelists with the recommended changes. The panel reviewed the modifications and approved the test stimuli in this round of the Delphi survey.

### 3.4 Phase 4

The differential diagnostic criteria were developed based on the inputs obtained from the expert panel based on their clinical expertise. Each of their opinions was considered, and the feature most suggested was ranked top in the list of differential features, and likewise, the features were orderly arranged. The common outcome that emerged from all these helped give the screening test a better outline. All ten participants contributed to the Delphi process resulting in a 100% response rate. Many of the features which the experts identified were overlapping and thus removed. Table **6** represents each of the differential features and the consensus of the experts.

The first five diagnostic criteria (1-5 from Table **6**), which received the highest consensus among the rankings provided by the experts from this list, were considered for identifying stuttering among school-going children.

## 4 DISCUSSION

The researchers in the field of child fluency disorders have emphasized the need for assessment tools to identify stuttering among school-going children which are performance-based, can be administered on one to one basis and are robust to note various speech and non-speech behaviors relevant to stuttering [27]. A recent study in this domain highlighted that most clinicians managing fluency concerns among school-going children solely rely upon SSI-4, while some choose the test of childhood stuttering [28]. Both these tests are widely used but pose certain limitations with respect to the suitability of the speech tasks with respect to the context and culture of the population being tested. The expert panel of the present study echoed similar opinions stressing the need to develop a culture-specific and performance-based screening tool for stuttering in school-age children. Further, the experts also highlighted that the proposed tool should be able to elicit speech samples of the children across several speaking conditions relevant to them. Accordingly, the tool incorporated speaking situations like reading, picture description and spontaneous speech-based narration. The choice of these speaking situations is in line with several other researchers who have emphasized that children’s fluency behaviors should be assessed in various situations, such as spontaneous speech, picture description and narratives which offer multiple speech samples to overcome the inconsistent nature of stuttering [19, 29, 30]. The length and quality of the speech sample have been identified as another factor that has to be taken care of during the assessment of stuttering [31]. Significantly, the speech samples collected for the fluency analysis are long and comprehensive enough (not just short phrases or sentences) because the richness of the sample could impact the detection of stuttering in children [32]. Similarly, the current instrument ensured that the situations and contexts selected for the reading, picture description, and narration activities were suitable for the study group in terms of culture, language, and educational grade and provided ample opportunities to generate a rich sample. The content validity stage also confirmed the effectiveness of the speech elicitation tasks through the strong agreement among the panel of experts.

Apart from the development of the tasks in the screening tool, another outcome of the present research was the differential diagnostic criteria which could aid in identifying children with stuttering from their normal speaking counterparts using the developed tasks. The study also identified the five most critical differential diagnostic criteria after the rank-based analysis of the expert opinions. These included sound and syllable repetition of more than 2%, presence of whole word repetitions of more than 5%, duration of prolongation instance to be more than 1 sec, effortful dysfluencies and presence of secondary behaviors. Most essential indicators used in the analysis align with the important speech and non-speech behaviors that multiple studies have identified as present during the onset of stuttering in childhood [19, 27, 29-32]. Thus, these criteria may provide a useful diagnostic value for the tool in distinguishing stuttering from age-appropriate normal disfluencies in school-aged children. This would enable prompt assistance for those who require it.

The Delphi method used in developing and validating the present tool has been observed to be useful in contributing to the successful development of clinically relevant assessment instruments [33-37]. This method involved gathering insights from Speech-Language Pathologists who specialize in stuttering. The goal was to establish a standard to differentiate between stuttering and regular disfluencies and determine if a child needs a more in-depth evaluation. By considering input from various experts, the screening test's pass/fail criteria became more objective.

## 5 CLINICAL IMPLICATIONS

In India, standardized screening tools for detecting stuttering in school-aged children are currently lacking. The existing tools are too lengthy for screening within a limited time frame. Our team has created a new screening tool that will allow clinicians to identify stuttering in this population quickly. This will aid in early detection and in gathering data on the prevalence of stuttering among school-aged children.

## CONCLUSION

The present study has aimed at offering a standardized screening tool for the presence of stuttering in school going population in India using a structured and systematic Delphi-based approach with the experts. This quick performance-based screening measure's culture, language and age-specific nature could help clinicians and researchers in epidemiological assessments. The tool can potentially promote early identification and intervention for stuttering in school-going children in an economical manner. Further studies examining the psychometric properties of the developed test are in progress.

## Figures and Tables

**Fig. (1):**
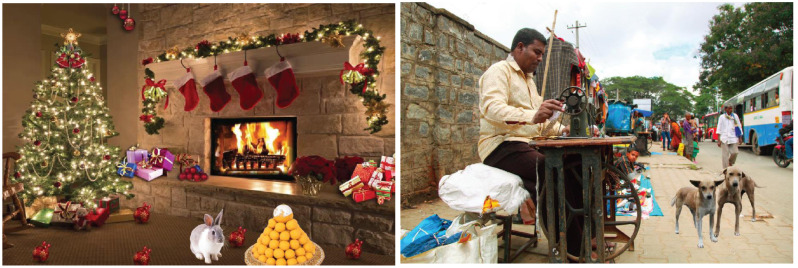
A sample of some of these pictures.

**Table 1:** Demographic details of SLPs.

**Demographic Details**	**Number of Participants**
Gender Male Female	4 1
Years of work experience 10-15 years	5
Working Context Academic Healthcare	5
Qualification Master’s Degree Doctoral Degree	1 4

**Table 2:** Demographic details of the participants.

**Characteristics**	**Number of Participants**
Gender Male Female	4 6
Years of work experience 5-10 Years 11-15 years 16-20 years	2 2 6
Working Context Academic Healthcare School University affiliated College	5 4 1
Qualification Master’s Degree Bachelor’s Degree with B.Ed.	6 4

**Table 3:** Demographic details of Speech-Language Pathologists.

**Demographic Details**	**Number of Participants**
Gender Male Female	5 5
Years of work experience 5-10 Years 10-15 years 15-20 years	2 2 6
Working Context Academic Healthcare Private practice	8 2
Qualification Master’s Degree Doctoral Degree	3 7

**Table 4:** Word count from each standard.

**Standard**	**No. of Words Selected**	**Examples of the Words**	**Examples of the Sentences used in the Reading Passage**
**4**	116	Big, come, out	It is very simple to include cleanliness in our habits.
**5**	1133	Bank, Petrol, Ticket	People who own pets recommend the same to everyone
**6**	850	Cough, Arrest, Judge	The scientist who first brought to light the facts about them was Galileo Galilei, a great scientist and astronomer.
**7**	624	Custom, Deal, Flourish	A working animal is an animal, usually domesticated, that is kept by humans and trained to perform tasks
**8**	902	Abuse, Debt, Domestic	Deforestation is the process of cutting trees to make space for industries and habitation for the ever-increasing human population.
**9**	675	Frank, Dew, Colonel	Politeness is the practical application of good manners or etiquette.
**10**	400	Credit, Dominate, Imprisonment	Denudation of the soil cover and subsequent washing down is known as soil erosion

**Table 5:** Spontaneous speech topics.

**Standard**	**Topics**
I	Health, Animals, and People Who Help Us
II	Grandparents, Best friends, Festivals in India
III	Sports, Under the Sea, Trees
IV	Cleanliness, Playground, Prizes
V	Types of Families, Swami Vivekananda, Talents
VI	Cricket, Nature, Seasons in the Year
VII	Gurudakshina, Netaji (Subhas Chandra Bose), Work is Worship
VIII	Freedom Fighters of India, Value of Time, Teamwork
IX	Nelson Mandela, the Importance of Having Good Manners, Unity in Diversity
X	Moral Acts, Waste Management in the Modern World, Consumerist Culture

**Table 6:** The consensus on the differential diagnostic criteria.

**S. No.**	**Differential Feature**	**Expert** **1**	**Expert** **2**	**Expert** **3**	**Expert** **4**	**Expert** **5**	**Expert** **6**	**Expert** **7**	**Expert** **8**	**Expert** **9**	**Expert** **10**
1.	Sound and syllable repetition of more than 2%	√	√	-	√	√	-	√	√	√	√
2.	Presence of whole word repetitions-more than 5%	√	√	√	√	-	√	√	√	√	-
3.	Duration of prolongation instance can be more than 1 sec	-	√	√	-	√	-	√	-	√	√
4.	Effortful dysfluencies	√	-	√	√	-	√	√	√	-	-
5.	Presence of secondaries	√	√	-	-	-	-	√	-	√	√
6.	More than 2 iterations in each repetition	-	√	-	√	√	-	-	√	-	-
7.	Speed of repetition greater than the rate of speech	√	-	√	-	-	-	√	-	-	√
8.	Presence of silent pauses within a word	-	√	√	-	√	-	-	-	-	-
9.	Presence of phonemic consistency	-	√	-	-	-	-	-	√	-	-
10.	Loci of stuttering in the initial word of a sentence	-	√	-	-	-	-	-	-	-	-
11.	Sudden termination of prolongation	-	-	-	-	-	√	-	-	-	-
12.	Unusual long hard contacts accompanied by the arrest of airflow or voicing	-	-	-	-	-	-	-	-	-	√

## Data Availability

All the data and supporting information are provided within the article.

## LIST OF ABBREVIATIONS

SLPs: = Speech Language Pathologists
SSI: = Stuttering Severity Instrument

## APPENDIX A

**Table:**

Sl. No.	-	Strongly Disagree	Disagree	Neutral	Agree	Strongly Agree
**Reading Task**		-	-	-	-	-
1	Are the target words and sentences suitable for the purpose of fluency assessment in each particular standard?	-	-	-	-	-
2	Are the words familiar/readable to children studying in a particular standard?	-	-	-	-	-
3	Does the passage contain age appropriate vocabulary for each particular standard?	-	-	-	-	-
4	Is the grammatical markers age appropriate?	-	-	-	-	-
6	Is the passage culturally suitable for children studying in each standard?	-	-	-	-	-
**Picture Description Task**		-	-	-	-	-
7	Are the pictures culturally suitable for children in different standard?	-	-	-	-	-
8	Is there continuity of information in a given picture card?	-	-	-	-	-
9	Is the picture card serving the purpose of eliciting maximum target words?	-	-	-	-	-
**Spontaneous Speech Tasks**		-	-	-	-	-
10	Are the topics selected appropriate for a particular standard?	-	-	-	-	-
11	Are there enough opportunities to speak in a given topic?	-	-	-	-	-
12	Do you have any suggestions in general?					
